# THE RAISE COUNCIL'S WORK IN DEVELOPING A NATIONAL FAMILY CAREGIVING STRATEGY

**DOI:** 10.1093/geroni/igac059.1133

**Published:** 2022-12-20

**Authors:** Salom Teshale

**Affiliations:** National Academy for State Health Policy, Washington, District of Columbia, United States

## Abstract

Since 2019, the RAISE Family Caregiver Advisory Council (FCAC) has met regularly to carry out its work of developing a national family caregiving strategy. This strategy incorporates five goals to support family caregivers, and key actions that a range of stakeholders can carry out centered around these goals. This overview will describe the RAISE FCAC’s work in developing the national family caregiving strategy, and highlight the development of recommendations and key actions to support the fourth goal, “Family caregivers’ lifetime financial and employment security is protected and enhanced.” This goal’s recommendations include supporting caregivers through flexible workplace policies; supporting affordable long-term services and supports; supporting financial education and planning; and reducing overall negative financial impacts of caregiving short and long-term.

